# Comparison of customized 3D-printed prosthesis and screw-rod-cage system reconstruction following resection of periacetabular tumors

**DOI:** 10.3389/fonc.2022.953266

**Published:** 2022-10-11

**Authors:** Dongze Zhu, Lei Wang, Jun Fu, Zheng Guo, Zhen Wang, Hongbin Fan

**Affiliations:** ^1^ Department of Orthopedic Surgery, Xi-jing Hospital, Fourth Military Medical University, Xi’an, China; ^2^ Department of Orthopedic Surgery, Tangdu Hospital, Fourth Military Medical University, Xi’an, China

**Keywords:** periacetabular tumors, hemipelvic reconstruction, screw-rod-cage system, 3D-printed, prostheses

## Abstract

**Background and purpose:**

Various operative methods are used for reconstructing pelvic girdle after resection of primary malignant periacetabular tumor has been reported. The objective of this study was to evaluate the accuracy, effectiveness, and safety of customized three dimensional-printed prosthesis (3DP) in the reconstruction of bone defects compared with conventional reconstruction using the screw-rod-cage system.

**Methods:**

A retrospective case–control analysis of 40 patients who underwent pelvic tumor resection and reconstruction with a customized 3D-printed prosthesis (3DP), or screw-rod-cage system (SRCS) between January 2010 and December 2019 was performed. The minimum follow-up time for patients alive was 2 years. Blood loss, operation time, complications, surgical margin, local recurrence, distant metastases, status at time of latest follow-up, MSTS-93 score, Harris hip score, and postoperative radiographic parameters were recorded. Moreover, overall survival, tumor-free survival, and prosthesis survival rates in both groups were compared.

**Results:**

Customized 3DP reconstruction was performed in 15 patients, and SRCS reconstruction was done in 25 patients. The group of patients treated with customized 3DP reconstruction had significantly shorter operation time (323.7 ± 83.7 vs. 393.6 ± 98.8 min; P = 0.028) and more precise (all P < 0.05) radiographic reconstruction parameters than patients in the SRCS group. Fewer complications (P = 0.026), better MSTS score (P = 0.030), and better Harris hip score (P = 0.016) were achieved in the 3DP group. Furthermore, the survival rate of prosthesis was also significantly better in the 3DP group (P = 0.039). However, blood loss, surgical margin, local recurrence, distant metastases, and status at time of latest follow-up had no significant difference between two groups.

**Conclusion:**

Compared with the screw-rod-cage system reconstruction, the customized 3D-printed prosthesis reconstruction is equally safe and effective, but it is more accurate and time-saving and is associated with fewer complications.

## Introduction

With improvements in chemotherapy and advances in imaging and surgical techniques, limb salvage surgery rather than aggressive amputation has been the mainstream treatment for patients with periacetabular tumors. Since the acetabulum is crucial for weight-bearing, limb-length, and hip joint function, reconstruction to restore acetabular stability after resection of periacetabular tumor is a worthwhile procedure (1–3). At present, multiple methods including hip transposition (4, 5), pedicle screw-rod system (6–8), biologic grafts (9, 10), and various endoprostheses (11–14) have been applied following pelvic tumor resection. However, due to the undesired complications of each method, the ideal reconstruction is still controversial.

We previously reported the patients with primary malignant pelvic tumor underwent resections and reconstructions with a screw-rod-cage system (SRCS) augmented with antibiotic cement. The results indicated that it was a relatively acceptable method with a lower complication rate, satisfactory functional outcomes, and feasibility for reconstruction for any type of periacetabular tumor resection (15). Despite this, the incidence of structural failures such as loosening and breakage of internal fixation has been reported to be between 11% and 33% (7, 15–18). In recent years, with the application of 3D printing technology in orthopedics, 3D-printed prosthesis (3DP) has been used for reconstructing pelvic bone defect after tumor resection. It can particularly enhance the precision of reconstruction, which is attributed to personalized manufacturing of the shape of prosthesis. The prosthesis also has excellent osteo-integration due to good biocompatibility caused by a porous structure at the interface, which might reduce the mechanical complications (19, 20).

To our knowledge, comparison of conventional SRCS and customized 3DP for pelvic reconstruction was rarely reported. The aim of this study was to evaluate the accuracy, effectiveness, and safety of these two reconstruction techniques after periacetabular tumor resection.

## Materials and methods

### Study design and participants

A retrospective case–control study, after being approved by the ethics committee of the authors’ hospital, was performed on patients with primary periacetabular sarcoma. A total of 40 patients (20 men and 20 women), who all signed the written informed consent, underwent periacetabular tumor resection and reconstruction with customized 3DP and SRCS from January 2010 to December 2019. There were 15 reconstructions with customized 3DP (group 1) and 25 with SRCS (group 2). According to the Enneking pelvic zoning classification system (21), the oncological zones of patients in the two groups were as shown in **Tables 1**, **2**.

**Table 1:** Patient demographics and clinical data in the 3DP group.

Case	Sex	Age (years)	Diagnosis	Resection type	Tumor size (cm)	Follow-up (months)	Operation time (min)	Blood loss (ml)	Surgical margin	MSTS, (%)	HHS	DD1 (mm)	DD2 (mm)	DA1 (°)	DA2 (°)	Complications	Oncological outcome	
																	Local recurrence	Distant metastases
1	M	53	Chondrosarcoma	II+III	7.0	112 (AWD)	360	5,500	Wide	70.0	70	3.4	2.2	2.2	2.4	No	Yes	No
2	F	65	Malignant giant cell tumor of bone	II+III	6.4	17 (DOD)	220	2,200	Wide	73.3	77	9.8	3.8	3.0	4.6	No	No	Lung
3	M	42	Malignant giant cell tumor of bone	II+III	13.8	75 (NED)	360	1,100	Marginal	76.7	83	2.2	5.6	3.3	3.3	No	No	No
4	F	6	Ewing’s sarcoma	I+II	5.0	57 (NED)	365	300	Wide	90.0	91	9.9	3.4	7.2	2.6	No	No	No
5	F	27	Chondrosarcoma	II+III	12.0	48 (NED)	270	2,000	Wide	83.3	86	1.6	2.1	1.9	2.6	No	No	No
6	F	43	Chondrosarcoma	II+III	11.0	7 (DOD)	410	3,900	Wide	80.0	84	5.9	3.3	1.0	1.5	No	Yes	No
7	M	59	Chondrosarcoma	II+III	5.9	39 (NED)	270	4,000	Wide	56.7	59	1.7	4.0	4.3	2.3	Deep infection	No	No
8	F	22	Chondrosarcoma	I+II+III	12.6	28 (NED)	315	3,400	Wide	83.3	89	1.5	8.2	4.1	3.5	No	No	No
9	M	20	Osteosarcoma	I+II	5.9	27 (NED)	415	4,700	Wide	93.3	94	4.0	3.2	2.4	1.3	No	No	No
10	F	8	Ewing’s sarcoma	I+II	8.5	26 (NED)	135	600	Wide	93.3	93	3.1	2.6	4.2	3.8	No	No	No
11	F	55	Chondrosarcoma	II+III	11.5	4 (DOD)	300	2,000	Marginal	80.0	83	6.4	2.2	6.3	3.2	No	No	Liver, pelvic
12	F	36	Chondrosarcoma	II+III	6.0	29 (NED)	480	5,400	Wide	90.0	90	4.1	4.7	3.3	4.5	No	No	No
13	F	37	Chondrosarcoma	II+III	9.5	16 (DOD)	340	5,000	Wide	83.3	82	7.6	3.5	4.2	3.5	Wound dehiscence	No	Liver
14	M	63	Chondrosarcoma	II+III	6.0	28 (NED)	310	4,700	Wide	86.7	80	6.2	4.7	3.9	3.1	No	No	No
15	M	64	Chondrosarcoma	I+II+III	15.0	24 (NED)	305	6,400	Marginal	83.3	84	1.4	6.8	6.1	2.6	No	No	No

NED, no evidence of disease; DOD, died of disease; AWD, alive with disease.

**Table 2:** Patient demographics and clinical data in the SRCS group.

Case	Sex	Age (years)	Diagnosis	Resection type	Tumor size (cm)	Follow-up (months)	Operation time (min)	Blood loss (ml)	Surgical margin	MSTS, (%)	HHS	DD1 (mm)	DD2 (mm)	DA1 (°)	DA2 (°)	Complications	Oncological outcome	
																	Local recurrence	Distant metastases
1	F	39	Chondrosarcoma	II+III	10.5	136 (NED)	390	5,400	Wide	76.7	76	6.2	9.4	9.3	6.3	Deep infection, internal fixation loosening	No	No
2	M	30	Chondrosarcoma	II+III	8.0	24 (DOD)	450	4,000	Wide	83.3	86	8.1	5.7	8.1	5.3	No	Yes	No
3	M	39	Chondrosarcoma	II+III	6.0	94 (DOD)	335	2,400	Wide	63.3	64	10.3	8.1	7.7	7.7	Internal fixation loosening	Yes	No
4	M	20	Ewing’s sarcoma	I+II	8.0	116 (NED)	300	3,300	Wide	90.0	90	9.6	9.8	5.2	3.9	No	No	No
5	F	38	Osteosarcoma	I+II	13.0	12 (DOD)	290	2,600	Wide	80.0	76	5.6	7.3	8.5	6.2	No	Yes	Lung
6	F	23	Plasma cell tumor	II+III	7.0	116 (NED)	240	3,000	Wide	86.7	90	13.1	8.5	9.1	4.5	No	No	No
7	F	36	Osteosarcoma	I+II	11.6	11 (DOD)	270	2,000	Marginal	90.0	90	6.7	0.6	6.7	4.0	No	Yes	Lung, skull
8	F	40	Osteosarcoma	II+III	15.0	33 (DOD)	340	4,000	Marginal	73.3	77	8.7	9.1	2.2	5.5	No	Yes	Lung
9	F	11	Ewing’s sarcoma	I+II+III	16.0	100 (NED)	525	5,200	Marginal	63.3	60	13.6	4.3	5.5	6.2	No	No	No
10	M	22	Ewing’s sarcoma	I+II	7.0	3 (DOD)	255	4,600	Wide	76.7	79	6.5	3.5	6.2	6.4	No	No	Lung, bone
11	M	23	Ewing’s sarcoma	I+II+III	15.0	5 (DOD)	410	3,400	Marginal	50.0	52	9.6	6.5	6.1	4.3	Dislocation	No	Lung
12	M	51	Chondrosarcoma	I+II+III	10.0	7 (DOD)	370	2,800	Wide	60.0	50	10.1	9.1	8.1	5.2	Deep vein thrombosis	Yes	No
13	F	16	Ewing’s sarcoma	II+III	8.0	2 (DOD)	385	3,000	Wide	80.0	71	3.8	5.3	1.7	5.9	Wound dehiscence	Yes	No
14	F	58	Chondrosarcoma	I+II+III	16.0	77 (NED)	430	2,000	Wide	66.7	68	4.8	4.8	4.7	8.2	Deep vein thrombosis	No	No
15	F	58	Chondrosarcoma	II+III	16.0	69 (NED)	685	7,250	Marginal	86.7	83	14.3	10.1	9.2	2.4	No	No	No
16	M	50	Chondrosarcoma	I+II+III	15.0	52 (NED)	405	7,000	Wide	70.0	78	8.8	6.0	5.7	6.4	Wound dehiscence, deep infection	No	No
17	M	46	Osteosarcoma	II+III	9.0	5 (DOD)	440	4,200	Wide	40.0	42	2.6	4.9	1.3	4.1	Dislocation	No	Lung
18	M	30	Chondrosarcoma	II+III	10.0	5 (DOD)	440	6,800	Wide	63.3	63	8.7	3.1	9.9	5.5	Dislocation	No	Skull
19	M	45	Osteosarcoma	II+III	11.0	5 (DOD)	380	5,000	Wide	76.7	83	2.4	3.8	1.6	5.2	Deep infection	Yes	Lung
20	M	39	Chondrosarcoma	II+III	13.3	20 (DOD)	530	4,000	Marginal	70.0	72	6.7	4.6	8.0	6.3	Internal fixation loosening	Yes	No
21	M	54	Chondrosarcoma	I+II	5.5	50 (NED)	345	3,500	Wide	80.0	79	2.3	3.7	10.6	3.7	No	No	No
22	M	51	Chondrosarcoma	I+II+III	12.8	3 (DOD)	410	4,000	Wide	73.3	73	7.0	4.4	2.5	4.3	No	No	Lung
23	M	54	Osteosarcoma	II+III	6.0	13 (DOD)	455	4,600	Wide	76.7	78	3.8	12.7	7.4	7.5	Wound dehiscence, deep infection	Yes	No
24	F	34	Osteosarcoma	II+III	10.0	38 (NED)	460	3,000	Wide	80.0	84	11.7	5.3	6.5	4.8	No	No	No
25	F	55	Plasma cell tumor	II+III	8.0	25 (NED)	300	2,200	Wide	76.7	74	12.7	5.9	7.1	7.6	No	No	No

NED, no evidence of disease; DOD, died of disease; AWD, alive with disease.

The inclusion criteria were as follows: (1) primary malignant tumors; (2) tumor extension into the acetabulum; (3) no major blood vessels, nerves, or organs involved; and (4) achieving expected surgical margins and good soft tissue coverage. The exclusion criteria were as follows: (1) secondary tumor or metastases; (2) reconstruction with other methods; (3) presence of distant metastases at the time of primary diagnosis; (4) incomplete clinical data.

In group 1, there were six men and nine women, with a mean age of 40.0 years (range, 6 to 65 years), and the mean maximum diameter of the tumor is 9.1 cm (range, 5.0 to 15.0 cm) (**Table 1**). In group 2, there were 14 men and 11 women, with a mean age of 38.5 years (range, 11 to 58 years), and the mean maximum diameter of the tumor is 10.7 cm (range, 5.5 to 16.0 cm) (**Table 2**). All patients with osteosarcoma and Ewing’s sarcoma received neoadjuvant chemotherapy.

### Surgical procedures

Surgical procedures detailing the acetabular approach and periacetabular resection were similar to those previously described in our reports (22, 23). According to the location of the tumor, patients were positioned in a left or right decubitus position. An extended ilioinguinal and iliofemoral approach was used. The external iliac vessels and femoral nerve were exposed, dissected, and protected. Depending on the tumor location and the degree of invasion, the tumor was en bloc resected in the normal muscle cuff.

**Figure 1:**
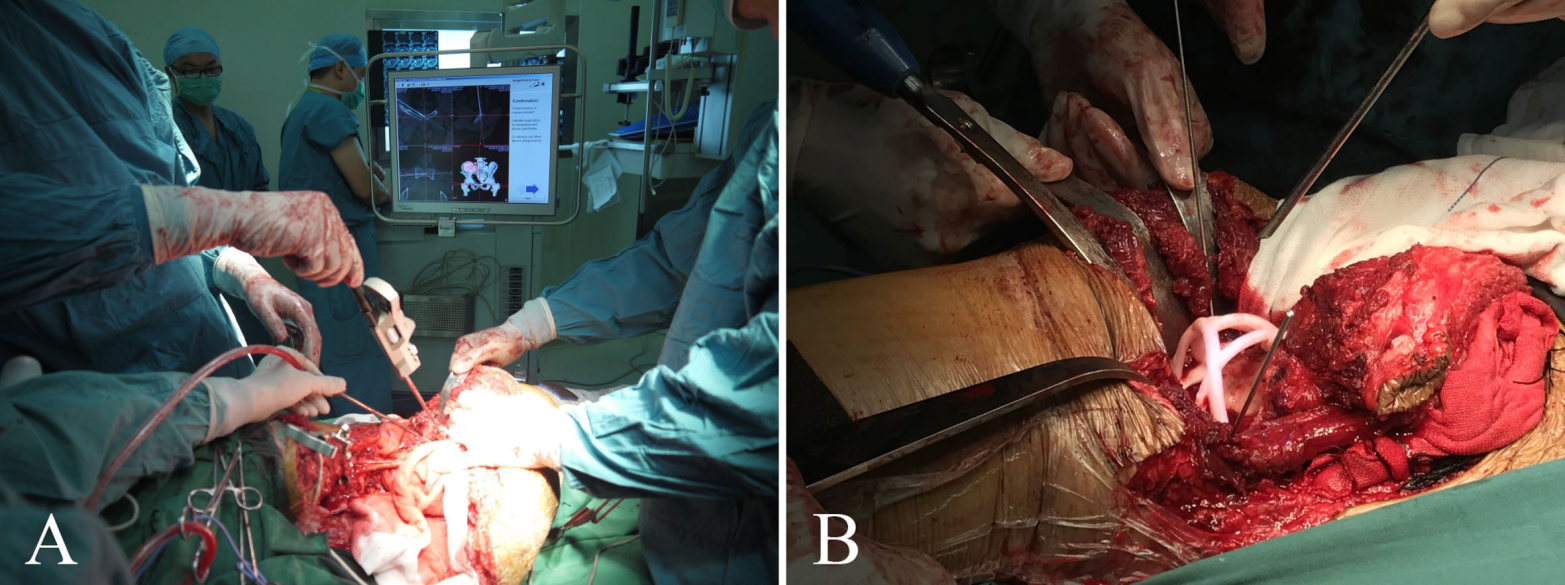
**(A)** Intraoperative application of the computer-assisted navigation system (CANS). **(B)** Intraoperative osteotomy was performed with the help of a 3D-printed osteotomy guide plate.

**Figure 2:**
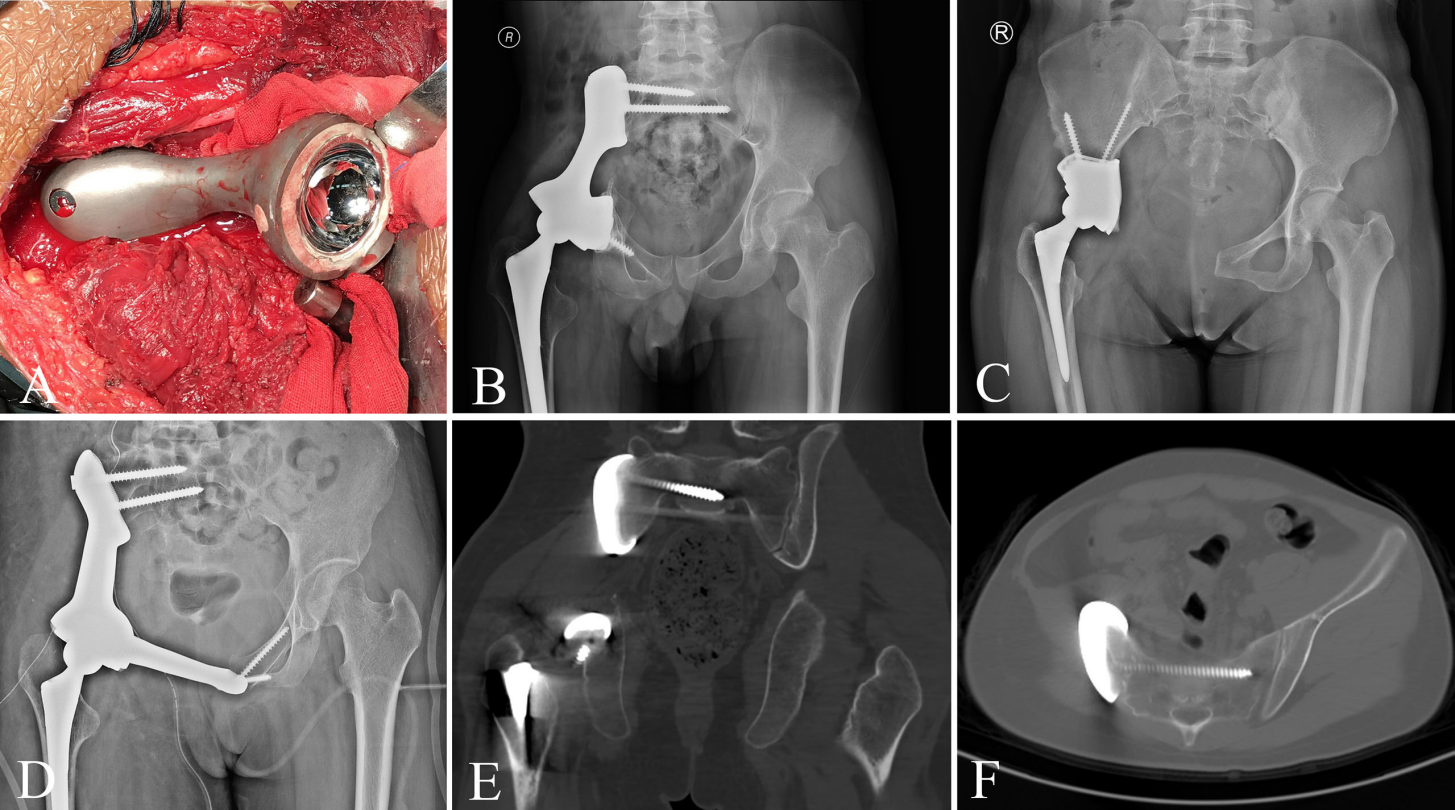
**(A)** The customized 3D-printed prosthesis was implanted following tumor resection. **(B–D)** Pelvic radiograph after 3D-printed prosthesis reconstruction. **(E, F)** CT scan shows that the bone is tightly bound to the prosthesis.

**Figure 3:**
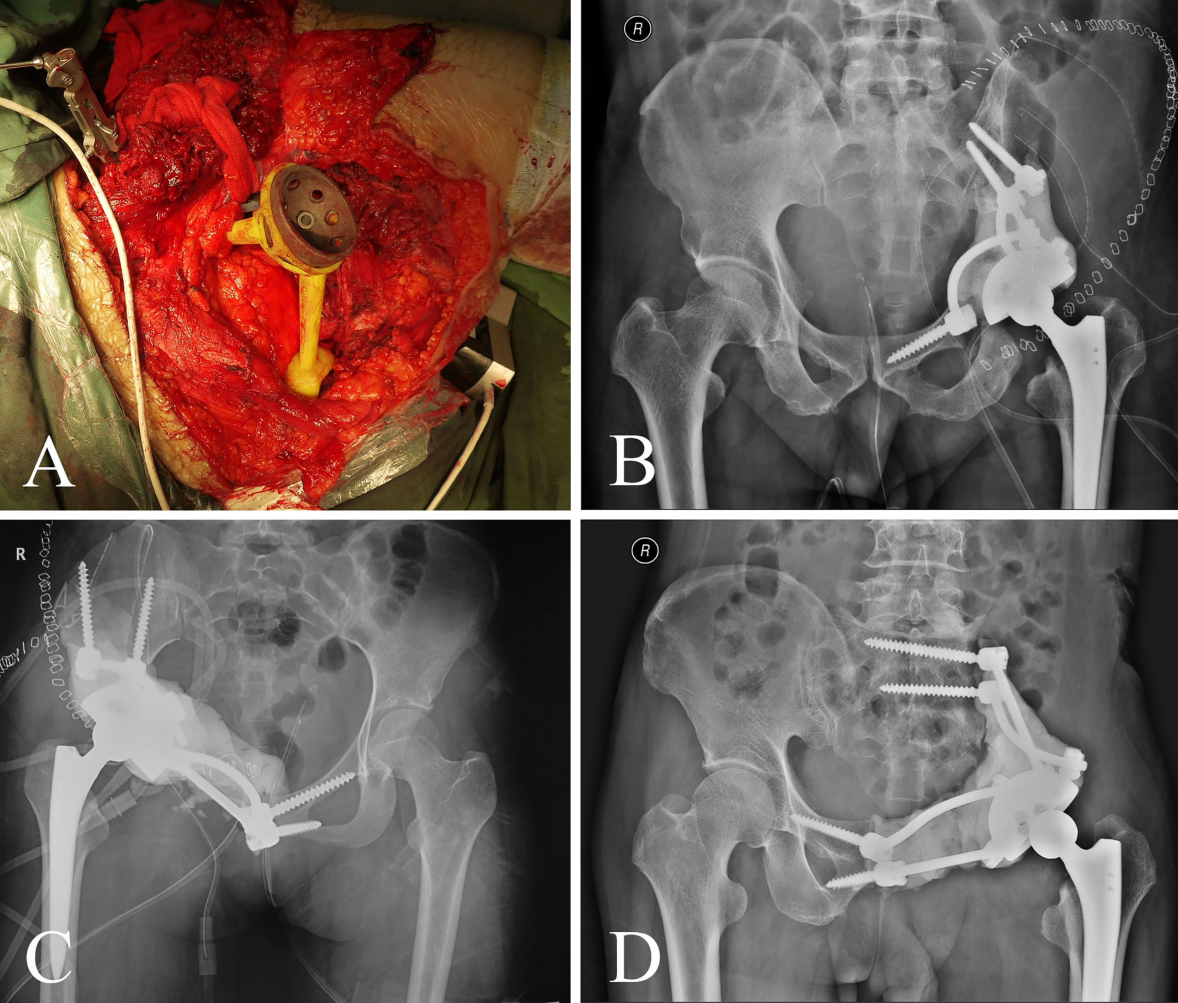
**(A)** The screw-rod-cage system was implanted after tumor excision. **(B–D)** Postoperative pelvic radiograph of screw-rod-cage system reconstruction.

Patients in group 1 received precise resection with the aid of a computer-assisted navigation system (CANS; Stryker Pacific, Ltd., Hong Kong, China) and a 3D-printed osteotomy guide plate (**Figure 1**). After careful removal of the tumor, the microporous surface of the customized 3DP was intimately pressed to the residual bone to reconstruct the pelvic ring as planned preoperatively (**Figure 2**). The detailed design and manufacture procedure of 3DP was described in our previous report (22). It took about 5 to 7 work days from design to the prosthesis availability. Moreover, 3DP costs approximately 1.5 times as much as SRCS. Patients in group 2 received precise resection, and an acetabular cage was placed at the planned position following connection with rods and screws assisted with CANS (**Figure 3**). Thereafter, bone cement was wrapped around the implant to reinforce the initial stability.

An intravenous antibiotic (cefazolin, 1 g/day) was usually administered during the first week after surgery to prevent infection. The administering antibiotic prophylaxis could be extended depending on the medical comorbidities, drainage tube retention time, and potential wound healing problems. After surgery, patients were requested to undergo non-weight-bearing activities for the first 6–8 weeks. Partial weight-bearing activities were encouraged between weeks 9 and 12, and full weight-bearing ambulation was allowed after 12 weeks.

### Follow-up and evaluation parameters

All patients completed the scheduled follow-up. They were assessed at 2 weeks, 1 month, and 3 months postoperatively; every 3 months for the first 2 years; and then every 6 months during 3 and 5 years, every year thereafter. Physical and radiological examinations were performed with every visit, and chest CT was obtained every 3 months for the first 2 years and thereafter every 6 months to detect the metastatic disease. Functional outcomes of patients were determined using the Musculoskeletal Tumor Society (MSTS) score 93 system and Harris hip score.

The outcome measures included blood loss, operation time, complications, surgical margin, local recurrence, distant metastasis, and radiographic parameters of reconstruction accuracy. Complications included wound dehiscence, dislocation, deep infection, internal fixation loosening, and deep vein thrombosis. Local recurrence and distant metastasis were assessed at follow-up as scheduled.

### Radiographic assessment

**Figure 4:**
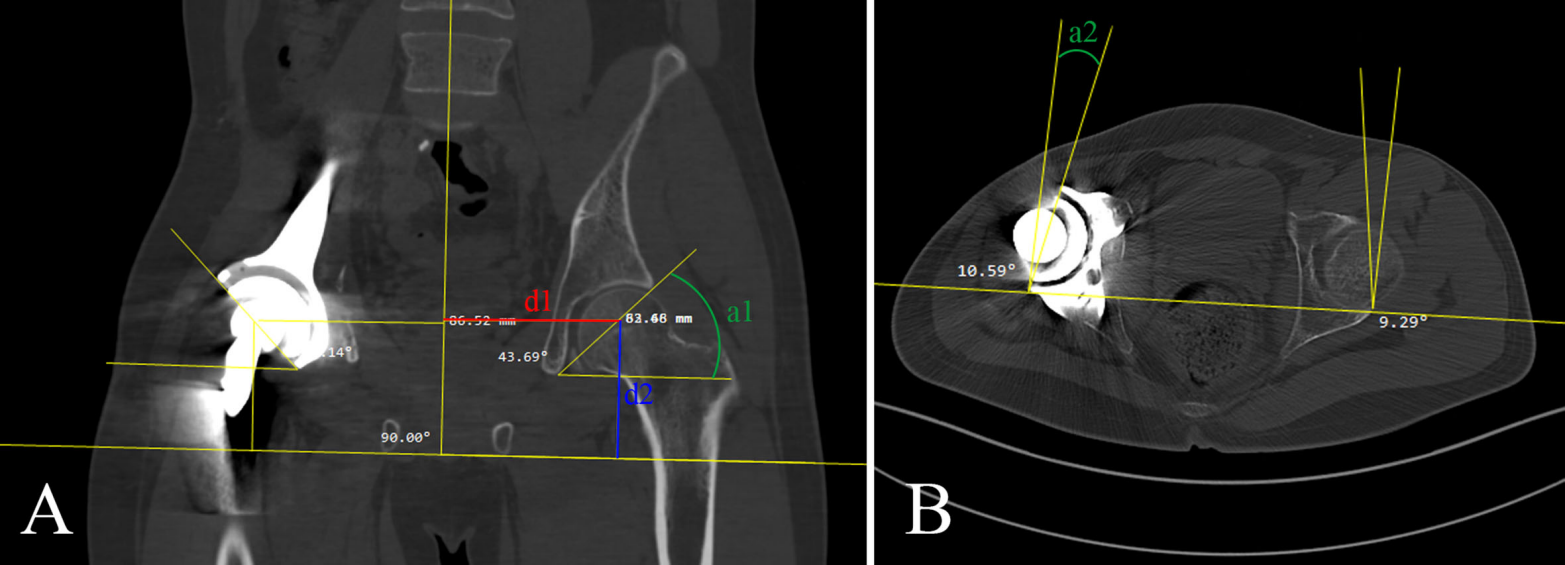
**(A)** Femoral head horizontal offset (FHO), femoral head vertical offset (FVO), and acetabular abduction angle (AAD) was shown in red line (d1), blue line (d2), and green line (a1), respectively. **(B)** Acetabular anteversion angle (AAV) was shown in green line (a2).

The evaluation methods drew from several classic assessment methods (24, 25). Four parameters were used to evaluate the accuracy of reconstruction: femoral head horizontal offset (FHO) indicated the distance from the rotation center of the femoral head to the central axis of the body (d1 in **Figure 4**). Femoral head vertical offset (FVO) indicated the vertical distance from the rotation center of the femoral head to the horizontal line passing the lowest point of the ischial tuberosity (d2 in **Figure 4**). Acetabular abduction angle (AAD) indicated the angle between the horizontal line and the line from the lateral superior acetabular margin to the inferior acetabular margin on the coronal CT image (a1 in **Figure 4**). Acetabular anteversion angle (AAV) indicated the angle between a line connecting anterior and posterior acetabular ridges and a line perpendicular to the line connecting the posterior pelvic margins on the transverse CT image (a2 in **Figure 4**). The above indicators were measured from CT images. The accuracy of the prosthesis placement was detected by comparing the difference of FHO, FVO, AAD, and AAV between the affected side and contralateral side in group 1 and group 2.

### Statistics

Continuous variables, including age, maximum diameter of the tumor, blood loss, operation time, duration of follow-up, MTST-93 score, Harris hip score, and postoperative radiographic measurement, were recorded as means with standard deviations. The *T*-test was used for parametric data and the Mann–Whitney U test for nonparametric data. Categorical variables, including sex, pathological diagnosis, resection type, complications, surgical margin, local recurrence, distant metastases, and status at time of latest follow-up, were expressed as counts with percentages and analyzed with Pearson chi-square test or Fisher’s exact test. The endpoint event for overall survival was death; the endpoint events for prosthesis survival were aseptic loosening, structural failure, and infection; and the endpoint events for tumor-free survival were local recurrence, metastasis, and death. The Kaplan–Meier calculations were used to evaluate overall survival, tumor-free survival, and prosthesis survival rates in both groups. The differences in the survival rate of both groups were compared with the log-rank test. Statistical analysis was performed using SPSS 25.0 (IBM), with the level of significance value defined as p < 0.05.

## Results

### Demographic and clinical data comparison

All patients received periacetabular tumor resection and reconstruction. There were 21 (52.5%) cases of chondrosarcoma, eight (20.0%) cases of osteosarcoma, seven (17.5%) cases of Ewing’s sarcoma, two (5.0%) cases of malignant giant cell tumor of bone, and two (5.0%) cases of plasma cell tumor in this study.

Baseline data are presented in **Table 3**, and there was no significant difference regarding age, sex, maximum diameter of the tumor, pathological diagnosis, and resection type between two groups (all P > 0.05). The median follow-up duration was 28 months for group 1 (range, 4 to 112 months) and 24 months for group 2 (range, 2 to 136 months). No patient was lost to follow-up, and the follow-up time of the two groups was similar (P = 0.605). There was similar blood loss between the two groups (3,413.3 ± 1,928.3 vs. 3,970.0 ± 1,497.8 ml, P = 0.314). The operation time in group 1 was significantly shorter than that of group 2 (323.7 ± 83.7 vs. 393.6 ± 98.8 min in P = 0.028, **Table 3**).

**Table 3:** Baseline, operative, and follow-up data of patients with pelvic tumor resection and reconstruction.

	Group 1 (n = 15) 3DP	Group 2 (n = 25) SRCS	p value
Sex*			0.327^a^
Male	6 (40.0)	14 (56.0)	
Female	9 (60.0)	11 (44.0)	
Age^†^ (year)	40.0 ± 20.0	38.5 ± 13.8	0.778^b^
Tumor size^†^ (cm)	9.1 ± 3.3	10.7 ± 3.5	0.152^b^
Pathological diagnosis*			0.328^c^
Chondrosarcoma	10 (66.7)	11 (44.0)	
Osteosarcoma	1 (6.7)	7 (28.0)	
Ewing’s sarcoma	2 (13.3)	5 (20.0)	
Others	2 (13.3)	2 (8.0)	
Resection type*			0.898^c^
I + II	3 (20.0)	5 (20.0)	
II + III	10 (66.7)	14 (56.0)	
I + II + III	2 (13.3)	6 (24.0)	
Blood loss† (ml)	3413.3 ± 1928.3	3970.0 ± 1497.8	0.314^b^
Operation time^†^ (min)	323.7 ± 83.7	393.6 ± 98.8	0.028^b‡^
Duration of follow-up^†^ (month)	28 (4~112)	24 (2~136)	0.605^d^
Complications*	2 (13.3)	12 (48.0)	0.026^a‡^
Wound dehiscence	1 (6.7)	3 (12.0)	
Dislocation	0	3 (12.0)	
Deep infection	1 (6.7)	4 (16.0)	
Deep vein thrombosis	0	2 (8.0)	
Internal fixation loosening	0	3 (12.0)	
Surgical margin*			1.000^c^
Wide	12 (80.0)	19 (76.0)	
Marginal	3 (20.0)	6 (24.0)	
Local recurrence*	2 (13.3)	10 (40.0)	0.152^c^
Distant metastases*	3 (20.0)	9 (36.0)	0.477^c^
Status at time of latest follow-up*			0.066^c^
No evidence of disease	10 (66.7)	10 (40.0)	
Alive with disease	1 (6.7)	—	
Died of disease	4 (26.7)	15 (60.0)	
MSTS-93 score^†^ (%)	81.5 ± 9.7	73.3 ± 12.0	0.030^b‡^
Harris hip score^†^	83.0 ± 9.1	73.5 ± 12.6	0.016^b‡^
Postoperative measurement^†^			
DD 1 (mm)	4.59 ± 2.91	7.91 ± 3.55	0.004^b‡^
DD 2 (mm)	4.02 ± 1.75	6.26 ± 2.76	0.008^b‡^
DA 1 (°)	3.83 ± 1.71	6.36 ± 2.73	0.003^b‡^
DA 2 (°)	2.99 ± 0.95	5.50 ± 1.43	<0.001^b‡^

^†^The values are given as the mean and the standard deviation. *The values are given as the number of patients, with the percentage in parentheses. ^a^Chi-square, ^b^t test, ^c^Fisher’s exact test, ^d^Mann–Whitney U test. ^‡^Significant difference between groups.

### Oncological outcome

**Figure 5:**
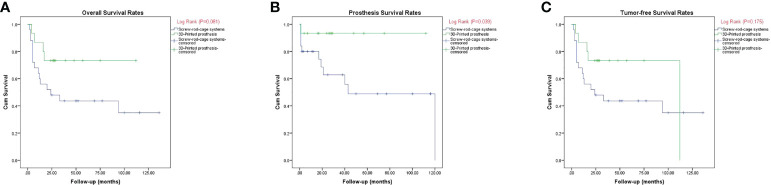
**(A–C)** Kaplan–Meier curves showing the overall disease survival **(A)**, prosthesis survival **(B)**, and tumor-free survival **(C)**.

A wide marginal resection was achieved in 12 patients (80.0%) in group 1 and 19 patients (76.0%) in group 2. Two cases (13.3%) of recurrence and three cases (20.0%) of metastasis were observed in group 1. Ten patients (40.0%) had recurrence and nine patients (36.0%) had metastasis in group 2. The rates showed no significant difference between the groups (P > 0.05). In group 1, 10 cases (66.7%) had no evidence of disease, one case (6.7%) was alive with disease, and four cases (26.7%) died of disease. In group 2, 10 cases (40.0%) had no evidence of disease and 15 cases (60.0%) died of disease. The Kaplan–Meier curves including overall survival, tumor-free survival, and prosthesis survival are shown in **Figure 4**. Regarding overall survival rate, there was no statistically significant difference between the groups (P = 0.081) (**Figure 5A**). With respect to prosthesis survival rate, it was significantly higher in group 1 than that in group 2 (P = 0.039) (**Figure 5B**). Additionally, there was no statistically significant difference regarding tumor-free survival rate between the two groups (P = 0.175) (**Figure 5C**).

### Complication

There was one patient with wound dehiscence and one with deep infection in group 1. On the contrary, wound dehiscence was observed in three cases, dislocation in three cases, internal fixation loosening in three cases, deep infection in four cases, and deep vein thrombosis in two cases in group 2 (**Table 3**). The complication rates were 13.3% in group 1 and 48.0% in group 2, respectively. Group 1 showed a significantly lower complication rate (P = 0.026).

### Functional outcome

The MSTS-93 score was 81.5 ± 9.7% (range, 56.7% to 93.3%) in group 1 and 73.3 ± 12.0% (range, 40.0% to 90.0%) in group 2. The Harris hip score was 83.3 ± 9.1 (range, 59 to 94) in group 1 and 73.5 ± 12.6 (range, 42 to 90) in group 2. Both MSTS-93 nd Harris hip score showed to be significantly higher in group 1 (P = 0.030, P = 0.016, **Table 3**). All patients were capable of being ambulatory without any aids.

### Radiographic evaluation

The difference in FHO between affected side and contralateral side (DD1) was 4.59 ± 2.91 mm in group 1, which was significantly lower compared with 7.91 ± 3.55 mm in group 2 (P = 0.004). The difference of FVO between affected side and contralateral side (DD2) was 4.02 ± 1.75 mm in group 1 and 6.26 ± 2.76 mm in group 2 (P = 0.008). The difference in AAD between affected side and contralateral side (DA1) was 3.83 ± 1.71 degree in group 1, which was significantly lower compared with the 6.36 ± 2.73 degree in group 2 (P = 0.003). The difference in AAV between affected side and contralateral side (DA2) also showed a statistically smaller degree (2.99 ± 0.95) in group 1 compared with the 5.50 ± 1.43 degree in group 2 (P < 0.001). Regarding the radiographic parameters aforementioned, all the differences between oncological side and contralateral side in group 1 were statistically less than those in group 2 (all P < 0.05, **Table 3**).

## Discussion

Resection of periacetabular malignancy is highly challenging due to its peculiar anatomy. Some authors resected periacetabular tumors without reconstruction. Although shorter surgical time and less blood loss were achieved, severe leg-length discrepancy was the commonest complication (26). The reconstruction requires more skilled techniques and detailed preoperative plans. Some prostheses such as saddle prosthesis (27), modular prosthesis (28), and ice-cream cone prosthesis (14, 29) have been reported with a varying degrees of success. However, the high incidence of complications demanded reconsideration of these conventional reconstruction techniques. Recently, the customized 3DP has shown promising early radiographic and functional results. It has been indicated for reconstruction of various parts of the body after tumor excision (30).

To promote the accuracy of reconstruction, CANS was introduced in oncological surgery (31–33). With the help of CANS, the customized 3DP can be perfectly fitted to the bone defect following tumor resection as well as possibly induce bone in-growth on the interface (20, 34–37). The technique of 3D printing in orthopedics could meet the need of customized, sophisticated, irregular morphology of artificial prosthesis, especially for massive bone defects caused by pelvic tumor resection (38). In the current study, we compared this 3DP reconstruction with previously reported screw-rod-cage reconstruction in patients with periacetabular malignancy. The results indicated that it was equally safe and effective but more accurate and time-saving.

In our study, the 3DP group showed significantly shorter operation time than the SRCS group, with an average time saving of 70 min. The surgical procedure was more time-saving in the 3DP group because of a preoperative meticulous plan and the precise matching between defect shape and morphology of prosthesis. To some extent, the precise prosthesis design was able to simplify the tedious procedures of intraoperative measurement, calibration, and adjustment for an appropriate implant position. Moreover, the 3D-printed osteotomy guide plate can shorten the operation time by improving the operating efficiency and osteotomy accuracy (19).

Meanwhile, radiographic evaluation showed that the 3DP group was significantly more accurate than the SRCS group in reconstruction (all P < 0.05). Inappropriate offset might result in limb-length discrepancy and in turn can lead to abnormal gait, low-back pain, instability, and patient dissatisfaction (39). Meanwhile, the AAD and AAV are both important positioning in acetabular reconstruction (40). The placement of acetabular components during surgery determines the accuracy of reconstruction and affects the stability of the prosthesis. In the SRCS group, the acetabular cup was guided by intraoperative CANS to adjust its position, while in the 3DP group, with the help of a simple and accurate installation process, the endoprosthesis can be installed in the precise position designed preoperatively. Postoperative radiographic evaluation also proved that 3DP reconstruction was more accurate.

In our study, two (13.3%) complications, namely, wound dehiscence and deep infection, were observed in 3DP reconstruction cases. The infectious case was treated with debridement and prosthesis removal. No aseptic prosthetic loosening or dislocation was found in the 3DP group. Other studies regarding 3DP reconstructions also showed a low complication rate. Xu et al. (20) reported promising outcomes after implanting 3D-printed personalized prosthesis in 10 patients with only one incision infection and zero prosthetic loosening or breakage. Similarly, Liang et al. (11) reported 35 patients with 3D-printed endoprosthesis after pelvic tumor resection and only two hip joint dislocations were observed. Our results were correlated with their findings, and the 3DP group had no structural complication. Zang et al. reported that the complication rate was 41.2% (7/17) by using a pedicle screw-rod system to reconstruct the defect after periacetabular tumor resection (41), which was similar to the results (48.0%, 12/25) in the current study. The screw-rod-cage system was embedded in bone cement to enhance initial stability, and the bone cement was susceptible to torsion, which might account for the high failure rates (42). During the follow-up, no aseptic loosening was observed in the 3DP group, which might be attributable to the porous structure at the interface of prosthesis. It could intimately contact the host bone through numerous canaliculi (35). Moreover, this structure was also reported to play a vital role in being an osteoconductive scaffold for bone in-growth, thereby stimulating bone formation on the junction of implant and bone (43). Theoretically, the osteointegration could considerably enhance the mechanical strength of the bone–implant interface and reduce the rates of prosthesis loosening.

In our study, the 5-year prosthesis survival rate of patients treated with 3DP (93.3%) was significantly better than that of SRCS (48.9%), which demonstrated a promising reconstruction procedure for periacetabular tumors. There was no structural complication in the 3DP group, and only one implant was removed due to deep infection. However, in the SRCS group, there were five cases of structural failure, three cases of deep infection, and two cases of recurrence that received hemipelvectomy resulting in prosthesis failure at 5 years. As mentioned previously, the difference in structural failure between two groups might be explained by the following reasons: 1) the porous structure of the 3DP at the interface might enhance the osteointegration and consequent stability; 2) the morphology of 3DP could well match the shape of the defect and acquire initial stability (35, 44). Additionally, there was no statistically significant difference with regard to overall and tumor-free survival rates between two groups. Demographic and pathological variables were not statistically different between the two groups (all P > 0.05). Moreover, the tumor was removed by the same surgical team following standard procedures. These might be the reasons why the recurrence and metastasis rates were not significantly different between groups. Therefore, compared to the conventional methods, the proposed technique in this study showed similar good disease control rates.

Nevertheless, our study did have some limitations. Firstly, this was a retrospective case–control study and the potential limitation could not be avoided like selection bias. Secondly, the number of patients in both groups was relatively small on account of the rarity of pelvic tumor in one orthopedic center. Future investigations with larger sample sizes are needed to further highlight the role of 3DP. Thirdly, the diverse tumor types and adjunctive therapy (chemotherapy) might have potential impact on the results. However, the primary purpose of this study was to compare the accuracy and effectiveness of the two reconstruction methods, and oncology outcomes were not really an issue here. Finally, the functional score of MSTS, although valid (45), was a relatively crude indicator in the current series. Therefore, the HHS score was also introduced to check the difference between groups. Despite these limitations, to our knowledge, there were few cohort studies that focused on the comparison of 3DP and other conventional reconstruction for managing peri-acetabular malignancy. Our institutional experience might add the information in this field.

To conclude, the customized 3DP reconstruction for the treatment of periacetabular tumors is equally safe and effective compared with SRCS reconstruction. Furthermore, it is more accurate and time-saving and is associated with fewer complications.

## Data availability statement

The original contributions presented in the study are included in the article/supplementary material. Further inquiries can be directed to the corresponding author.

## Ethics statement

The studies involving human participants were reviewed and approved by Teaching and Research Ethics Committee of Xi-Jing Hospital, Fourth Military Medical University. Written informed consent to participate in this study was provided by the participants’ legal guardian/next of kin.

## Author contributions

DZ, JF, ZG, ZW, and HF contributed to conception and design of the study. DZ and JF organized the database. DZ and LW performed the statistical analysis. DZ and LW wrote the first draft of the manuscript. DZ, LW, and JF have contributed equally to this work and share first authorship. All authors contributed to manuscript revision, read, and approved the submitted version.

## Funding

This work was supported by the National Key R&D Program of China (No. 2016YFB1101104) and Shaanxi Provincial Key R&D Program, China (No. 2018ZDXM-SF-075).

## Conflict of interest

The authors declare that the research was conducted in the absence of any commercial or financial relationships that could be construed as a potential conflict of interest.

## Publisher’s note

All claims expressed in this article are solely those of the authors and do not necessarily represent those of their affiliated organizations, or those of the publisher, the editors and the reviewers. Any product that may be evaluated in this article, or claim that may be made by its manufacturer, is not guaranteed or endorsed by the publisher.
